# Effects of Grape Pomace on Growth Performance, Nitrogen Metabolism, Antioxidants, and Microbial Diversity in Angus Bulls

**DOI:** 10.3390/antiox13040412

**Published:** 2024-03-28

**Authors:** Yingqi Li, Changxiao Shi, Jiajie Deng, Xinjun Qiu, Siyu Zhang, Huili Wang, Xiaoli Qin, Yang He, Binghai Cao, Huawei Su

**Affiliations:** 1 State Key Laboratory of Animal Nutrition and Feeding, College of Animal Science and Technology, China Agricultural University, Beijing 100193, China; liyingqi1230@alu.cau.edu.cn (Y.L.); scx1107@cau.edu.cn (C.S.); dengjj@cau.edu.cn (J.D.); s20213040648@cau.edu.cn (S.Z.); s20223040747@cau.edu.cn (H.W.); qinxiaoli@cau.edu.cn (X.Q.); he.yang@cau.edu.cn (Y.H.); caobh@cau.edu.cn (B.C.); 2 School of Tropical Agriculture and Forestry, Hainan University, Haikou 570228, China; qiuxinjun@hainanu.edu.cn

**Keywords:** grape pomace, nitrogen metabolism, antioxidant activity, microbiota, Angus bulls, digestibility

## Abstract

Polyphenol-rich grape pomace (GP) represents a valuable processing by-product with considerable potential as sustainable livestock feed. This study aimed to investigate the effects of different levels of GP on the growth performance and nitrogen utilization efficiency, antioxidant activity, and rumen and rectum microbiota of Angus bulls. Thirty Angus bulls were allocated three dietary treatments according to a completely randomized design: 0% (G0), 10% (G10), and 20% (G20) corn silage dry matter replaced with dried GP dry matter. The results showed that the average daily gain (ADG) of the G0 group and G10 group was higher than that of the G20 group (*p* < 0.05); urinary nitrogen levels decreased linearly with the addition of GP (linear, *p* < 0.05). In terms of antioxidants, the levels of catalase (CAT) in the G10 group were higher than in the G0 and G20 groups (*p* < 0.05), and the total antioxidative capacity (T-AOC) was significantly higher than that in the G20 group (*p* < 0.05). In addition, in the analysis of a microbial network diagram, the G10 group had better microbial community complexity and stability. Overall, these findings offer valuable insights into the potential benefits of incorporating GP into the diet of ruminants.

## 1. Introduction

Grape pomace (GP) predominantly comprises grape seeds, skins, and stalks, containing high levels of carbohydrates and proteins [1]. Specifically, grape seeds are abundant in polyunsaturated fatty acids, contributing to animal health when consumed in moderation. Additionally, GP is a rich source of polyphenols, known for their potent antioxidant properties [2,3]. With the increasing demand for wine and concerns over the environmental impact of the traditional treatments, there is a pressing need to explore efficient resource utilization strategies for GP.

Grape pomace is rich in diverse polyphenolic compounds, including tannins, proanthocyanidins, anthocyanidins, flavonoids, and phenolic acids, attributable to the extraction conditions, efficiency, and chemical reactions inherent in the wine-making process [1,4,5]. Voicu et al. [6] showed that the growth performance and digestive metabolism of beef cattle are not adversely affected by many of these polyphenolic compounds. Notably, condensed tannins (CT) stand out as the foremost bioactive constituents, constituting 58.7% of the total phenolic compounds in GP [7]. Condensed tannins have more hydroxyl groups and a higher polymerization of polyphenols, making them stronger antioxidants than simple phenolics [8]. Increasing evidence suggests that polyphenolic compounds can be utilized as dietary additives in adult ruminant diets [9,10]. Furthermore, the structure–activity relationships and chemical environment of the polyphenol compounds in GP affect the antioxidant efficiency and balance [11]. Studies have shown that feeding 25 kg lambs with 10% GP and feed increased the total antioxidant capacity (T-AOC) and Glutathione Peroxidase 4 (GPX4) and superoxide dismutase (SOD) activity in the lambs [12].

Condensed tannins have the ability to bind and precipitate proteins. Due to their complex structure and large relative molecular mass, hydrolysis of CT can occur only under strong oxidation and acidic conditions. Therefore, CT have a strong antibacterial ability, which can destroy microbial membranes by inhibiting the substrate of enzymes and inactivating membrane-binding proteins [13]. In a previous experiment [14], it was found that GP can improve the anti-inflammatory effect of Caco-2 cells. In addition, the addition of GP can inhibit the growth of pathogenic bacteria such as *Enterobacteriaceae* and *E. coli.* in feces [15].

In the late fattening period of beef cattle, long-term feeding with a high-concentrate diet can stimulate the release of proinflammatory cytokines, which leads to oxidative stress in the body [16]. We can use feed rich in polyphenols, and GP is one of the best choices; however, dietary polyphenols can have toxic effects on animals’ bodies when fed in large doses. The evidence indicates that dietary polyphenols can have adverse effects on animals’ bodies through their antioxidant action [17]. A high dose of GP may over-protect proteins and hinder the digestion and absorption of nutrients by reducing the activity of rumen microorganisms [8]. Therefore, this evidence suggests that although wine grains can be used as an antioxidant diet for ruminants, we need to explore the appropriate replacement ratio to prevent a high concentration reducing the stability of ruminal and rectal microbial communities, which is detrimental to the health of beef cattle.

At present, the animal studies on GP mainly focus on growth performance [6,7] and nitrogen utilization efficiency (NUE) [18], and the effects of GP on the antioxidant activity and health of beef cattle are still unclear. Therefore, the purpose of this study was to investigate the effects of different proportions of GP on the growth performance, antioxidant activity, and rumen and rectal microbiota of beef cattle through feeding experiments. The purpose is to understand the use of GP and provide a theoretical basis for its application in diets.

## 2. Materials and Methods

### 2.1. Animals, Diets, and Management

This experiment was carried out with permission from the Animal Care Committee (approval number: AW82113202-1-1), China Agricultural University. The present trial was conducted from February to June 2021 at Benwang Farm (Ningxia, China).

The grape pomace, sourced from wineries in Yinchuan City, Ningxia Hui Autonomous Region, consisted of Cabernet Sauvignon grape varieties. The GP underwent a drying process to achieve a less than 14% moisture content. Subsequently, it was crushed and sieved through a 10-mesh sieve. The resulting dried grape pomace (DGP) was stored in a dry and cool environment.

This study used 30 16-month-old Angus bulls with an initial body weight of 580.57 ± 42.72 kg (mean ± SD) as the experimental animals. All the cattle were randomly allocated 1 of 3 dietary treatments. The bulls were provided with a basal total mixed ration (TMR, Table 1), while the bulls in the G10 and G20 groups had the corn silage in the G0 group substituted with either 100 or 200 g/kg of DGP meal, respectively. Although DGP was used as a fiber substitute for corn silage and wheat bran, its content of other nutrients also differed (Table 2). The nutrient content of the TMR was formulated to meet the growth needs of the animals, as required by the NASEM (2016) [19].The experiment was performed over 141 days, including 12 days of adaptation and a 129-day experimental period. The average DMI was 12.87 kg/d throughout the trial period. Restricted feeding was designed to exclude the differences brought about by different feed intakes. All the groups were fed two times at 7:00 and 17:00. Drinking water was offered ad libitum.

### 2.2. Sample Collection

Before the start of the experiment, the initial live weights of the fasting bulls were taken. On the morning of the 129th day, the final live weights of each bull (before feeding) were taking using a weighbridge, and the average value was measured twice. The average daily gain (ADG) was calculated as the body weight gain (BWG) divided by the number of experimental days, and the feed conversion ratio (FCR) was calculated as DMI/ADG. Feed samples were collected from the 21st to the 23rd day of each month. On the 130th day, in the morning (after feeding) and in the afternoon, the urine and feces of each bull were collected. The urine samples were collected using a self-made collector and then shaken with a glass rod to retain about 60 mL of urine, 10 mL of 0.5 mol/L dilute sulfuric acid was added to prevent the volatilization of nitrogen, and they were stored at −20 °C until analysis. Feces samples were collected from the bull rectums using sterile polyethylene gloves. The feces were filled separately into two 2 mL cryotubes and stored with dry ice after sampling to determine rectal microorganisms. A total of 150 g of feces was collected from each bull and stored in a plastic bag to determine their apparent digestibility. Then, on the 131st and 132nd day, another 150 g of feces from each bull was collected at 6:00, 12:00, 18:00, and 24:00 to determine their apparent digestibility, and feces from the same bull were mixed into one sample per individual. On the morning of the 133rd day, 2 tubes of 5 mL blood samples were collected and placed in a 4 °C refrigerator for 3 h. Then, the supernatant was centrifuged at 3000× *g* and stored in dry ice. After the blood collection, rumen fluid was collected and filtered with four layers of gauze. The pH was determined immediately, two 2 mL cryotubes were divided for microbial analysis, and the rest was packed into two 10 mL centrifugal tubes to determine its ammonia nitrogen content. All the samples (feces, blood, and rumen fluid) stored on dry ice were stored at −80 °C. The feed and fecal samples were dried at 65 °C and ground through a 2 mm sieve for subsequent analysis.

### 2.3. Chemical Analysis

#### 2.3.1. Feed Analysis and Nutrient Digestibility

The neutral detergent fiber (NDF), acid detergent fiber (ADF), acid detergent lignin (ADL), and acid-insoluble ash (AIA) of the feed and rectum feces were determined using the method of Van Soest et al. [22], while the ether extract (EE), dry matter (DM), crude protein (CP), and ash in the feed and rectum feces were analyzed using the methods of AOAC [23]. Their apparent digestibility was determined using the AIA method [24]. The concentration of AIA was employed as an internal indicator to estimate the apparent total tract digestibility of various nutrients and quantify the fecal DM output. After thawing the urine samples (4 °C) and shaking them using a vortex oscillator, 5 mL of each sample was extracted and mixed, and the content of nitrogen in 1 mL of the urine was determined using the AOAC methods after the shaking [23]. A creatinine (Cr) assay kit (sarcosine oxidase; Nanjing Jiancheng Bioengineering Institute, Nanjing, China) was used to analyze the urine creatinine levels. Formula [25] calculates urine excretion by using urinary creatinine and body weight as an indicator. The total urinary nitrogen is the product of the total urine excretion and the nitrogen content in 1 mL of urine.

#### 2.3.2. Blood Antioxidant Indicators

Catalase (CAT), T-AOC, SOD, and malondialdehyde (MDA) were examined in triplicate using commercial test kits (Nanjing Jiancheng Institute of Biological Technology, Nanjing, China), except for ROS, the test for which was produced by Shanghai Mlbio Institute of Biological Technology (Shanghai, China).

#### 2.3.3. Rumen Fermentation Parameters and Feed Fatty Acids

The rumen fluid’s pH was measured using a portable pH meter (Testo AG, Schwarzwald, Germany). The concentrations of VFA in the rumen fluid and fatty acid in the diet were determined using a gas chromatograph (GC-2014, Shimadzu Corporation, Kyoto, Japan). A Tecan Sunrise microplate reader (Tecan, Mannedorf, Switzerland) was used to measure the NH_3_-N concentration in the rumen fluid.

#### 2.3.4. Microbiota Analysis

Using a TGuide S96 Magnetic Soil/Stool DNA Kit (TIANGEN Biotech Co., Ltd., Beijing, China), DNA was extracted and determined using the Qubit dsDNA HS Assay Kit and the Qubit 4.0 Fluorometer (Invitrogen, Thermo Fisher Scientific, Dallas, OR, USA). The 338F: 5′-ACTCCTACGGGAGGCAGCA-3′ and 806R: 5′-GGACTACHVGGGTWTCTAAT-3′ universal primer set was used to amplify the V3–V4 region of the 16S rRNA gene from the genomic DNA extracted from each sample. The complete sequencing dataset was accessed through the National Center for Biotechnology Information (NCBI) (accession number PRJNA1063547). During that time, QIIME2 (2020.6) and R software (4.3.0) were used to calculate and display the Alpha diversity. To test for significant taxonomic differences among groups, we also used the Linear Discriminant Analysis (LDA) effect size (LEfSe [26]). Logarithmic LDA scores of 3.5 were used to determine discriminative features.

### 2.4. Statistical Analysis

One-way analysis of variance (ANOVA) was conducted using a general linear model in SPSS (version 25.0) to determine significant differences among the samples. Mean values were compared using Tukey’s test. The level of statistical significance was set to *p* < 0.05. Microbial co-occurrence networks were built using the CoNet module (Spearman’s correlation > |0.7| and *p* < 0.05) in Cytoscape 3.9.1 and were viewed in Gephi software 0.10.1. Spearman’s correlation coefficient was used to evaluate the effects of the heatmap using RStudio (2023.06.0+421).

## 3. Results

### 3.1. Growth Performance

The growth performance and feed intake data are presented in Table 3. The initial live weights were not different across diets (*p* > 0.05). The elevated amount of GP added to the TMR resulted in a notable decline in the final live weights, displaying a linear trend (*p* = 0.054), whereby the G0 group and G10 group exhibited a significantly greater ADG compared to the G20 group (*p* < 0.05). Additionally, the inclusion of 20% DGP led to significantly higher FCR values (*p* < 0.05) in contrast to the other groups.

### 3.2. Nutrients’ Apparent Digestibility and Nitrogen Metabolism

The nutrients’ apparent digestibility is presented in Table 4. The apparent digestibility of EE was lower in the bulls fed the 20% dried GP diet compared to those fed the 10% dried GP and control diets (*p* < 0.05). No effect was seen among treatment groups on the apparent digestibility of DM, CP, NDF, and ADF (*p* > 0.05). Being fed a diet containing DGP decreased linearly the urinary nitrogen levels compared to the control diet (*p* < 0.05).

### 3.3. Blood Antioxidants

In terms of the antioxidant indicators (Table 5), the concentration of CAT in the serum of G10 was higher than that in the other two groups, and the content of T-AOC in the G10 group was higher than that of the G20 group (*p* < 0.05). No effect was seen among the treatment groups on the blood antioxidant levels (*p* > 0.05).

### 3.4. Ruminal Fermentation Parameters

The results on the rumen fermentation parameters among the dietary treatments are shown in Table 6. In terms of the VFA, the acetate-to-propionate ratio was increased after the 20% DGP diet group compared to those fed the 10% DGP and control diets (*p* < 0.05). The rumen valerate value decreased linearly with an increase in DGP supplementation (*p* < 0.05). No effect was seen on the other indicators (*p* > 0.05).

### 3.5. Microbiota

**Figure 1:**
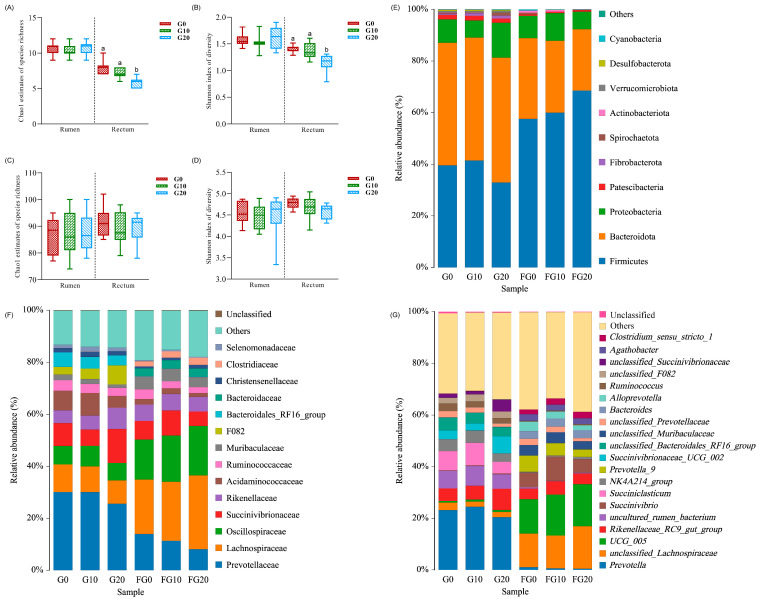
Microbial diversity and composition with dried grape pomace treatment at different levels in the rumen and rectum. Comparison of diversity metrics of microbial communities at the phylum (**A**,**B**) and genus (**C**,**D**) levels. Comparison of within-community richness (Chao1 estimates) (**A**,**C**) and diversity (Shannon’s index) of the microbiota of rumen and rectum among treatment groups (**B**,**D**). Box-whisker plots show average values of richness and diversity; boxes denote interquartile ranges with a line at the median; whiskers indicate minimal and maximal values; and error bars indicate the standard error for each treatment group. Different letters above bars indicate statistically significant differences between groups in (**A**,**B**) (*p* < 0.05). Microbial composition of different groups. Each bar represents the average relative abundance of each bacterial taxon within a group. (**E**) Taxa assignments at the phylum level. (**F**) Taxa assignments at the family level. (**G**) Taxa assignments at the genus level. G and FG indicate rumen and rectum. G0, the control group; G10, the 10% dried grape pomace treatment group; G20, the 20% dried grape pomace treatment group.

The total number of reads across all samples was 4,797,906, with an average of 79,775 reads per sample. Figure 1A–D compare the richness and diversity of the rumen microbiota among the three groups. There was no difference between the richness and diversity of the rumen microbiota (*p* > 0.05). At the phylum level (Figure 1A,B), the G20 group had decreased values in the microbial diversity indexes of Chao1 and Shannon compared with G0 (*p* < 0.05) and G10 (*p* < 0.05) in its rectal microbiota.

**Figure 2:**
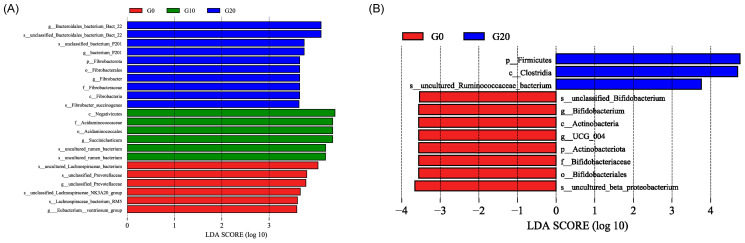
LDA scores for the bacterial taxa differentially abundant among G0, G10, and G20 (LDA > 3.5). (**A**) is the result in the rumen; (**B**) is the result in the rectum. Red bars indicate taxa were enriched in G0; green bars indicate taxa were enriched in G10; and blue bars indicate taxa were enriched in G20. G0, the control group; G10, the 10% dried grape pomace treatment group; G20, the 20% dried grape pomace treatment group.

A total of 14 phyla were observed, with the microbial top 10 shown in Figure 1E. The relative sum of Bacteroidota, Proteobacteria, and Firmicutes accounted for over 90% of the total abundance of the rumen and rectal microbiota. Increasing the level of GP linearly increased the relative abundance of the Firmicutes phylum (*p* = 0.001) and linearly decreased the relative abundances of Bacteroidota and Actinobacteriota (*p* < 0.05) in the rectum (Figure 2B; Supplementary Table S1). Additionally, the relative abundance of Desulfobacterota had a quadratic relationship with the level of GP (*p* < 0.05).

At the family level (Figure 1F; Supplementary Table S2), an increasing level of GP linearly increased the relative abundance of F082 (*p* < 0.05), while it linearly decreased the relative abundance of Acidaminococcaceae (*p* < 0.05) in the rumen (Figure 2A; Supplementary Table S2). In the rectum, the relative abundances of Christensenellaceae, Lachnospiraceae, and Oscillospiraceae linearly increased (*p* < 0.05), whereas the relative abundances of Acidaminococcaceae and Prevotellaceae linearly decreased (*p* < 0.05; Supplementary Table S2).

At the genus level (Figure 1G; Supplementary Table S3), the relative abundance of *Succiniclasticum* had a quadratic relationship with the level of GP (*p* < 0.05) in the rumen (Figure 2A; Supplementary Table S3). In the rectum, an increasing level of GP linearly increased the relative abundance of *NK4A214_group* (*p* < 0.05), while it linearly decreased the relative abundance of *Prevotella* (*p* < 0.05).

**Figure 3:**
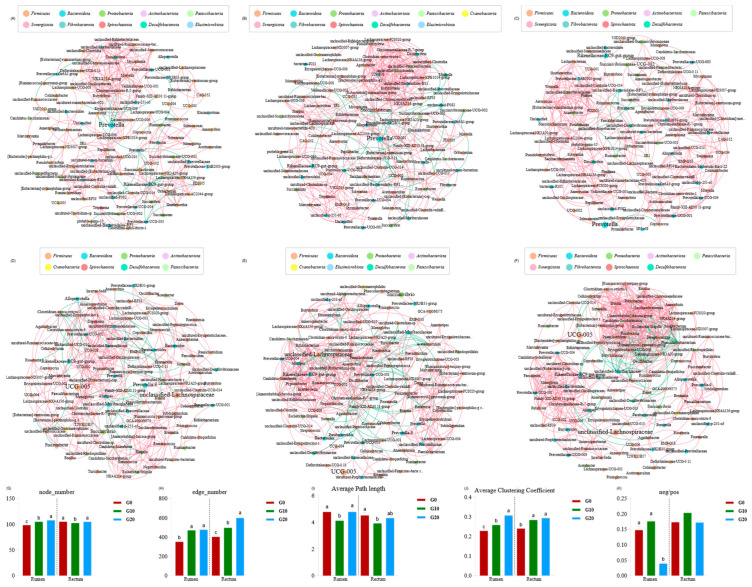
Interaction networks of rumen and rectum microbiota based on dried grape pomace at different levels. The 16S-rRNA-gene-based correlation network of the microbiota is calculated from the bacteria with a relative abundance greater than 0.2%. (**A**) is G0 rumen; (**B**) is G10 rumen; (**C**) is G20 rumen; (**D**) is G0 rectum; (**E**) is G10 rectum; (**F**) is G20 rectum. Nodes represent individual genera; edges represent significant positive Spearman’s correlations (ρ > 0.7; *p* < 0.05). Node size is scaled based on the overall abundance of each taxon in the microbiota. Red edge indicates positive correlations, and green edge indicates negative corrections. The numbers of nodes and edges and average path lengths and average clustering coefficients of rumen and rectum bacteria co-occurrence patterns (**G**–**J**). Neg/pos (**K**); the ratio of negative correlation to positive correlation. Different letters above bars indicate statistically significant differences between groups in (**G**–**K**) (*p* < 0.05). G0, the control group; G10, the 10% dried grape pomace treatment group; G20, the 20% dried grape pomace treatment group.

The interactions between the microorganisms in the rumen and rectum systems based on different proportions of GP rations are complex (Figure 3). The present study used a microbial network to identify keystone taxa that had a crucial impact on the microbial structure and to construct correlations among species. The node and edge numbers of the network were used as the topological parameters of the network to assess the bacterial network’s complexity. The higher the node and edge numbers, the more complex the network. The average path length and the average clustering coefficient represent the specificity of the network. Negative and positive correlation ratios (neg/pos) were used to assess the bacterial network’s stability, and the values were positively correlated with the network stability. Overall, the microbial communities in the rumen and rectum were more complex with the addition of GP, and both G10 and G20 had larger average clustering coefficients compared to G0; especially, G10 had a smaller average path length, suggesting the small-world characteristics of both treatment groups. Interestingly, the microbial community in G10 had a higher stability. Based on the network analysis, the dominant genera in the rumen *NK4A214-group* and *Butyrivibrio* in the G0 group, *NK4A214-group* and *unclassified-Christensenellaceae* in the G10 group, and *Pantoea* in the G20 group and the dominant genera in the rectum *UCG-002* and *UCG-009* in the G0 group, *UCG-002* and *UCG-009* in the G10 group, and *Paeniclostridium* in the G20 group were identified.

**Figure 4:**
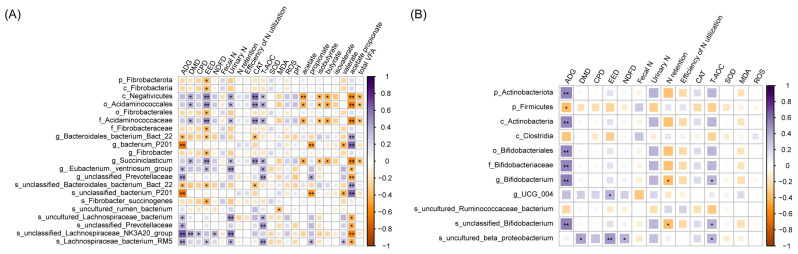
Correlation between apparent digestibility of nutrients; rumen fermentation parameters (rectum results exclude this indicator); nitrogen metabolism; and bacteria abundance in the rumen (**A**) and rectum (**B**). Only significant correlations and bacteria abundances > 0.5% are shown. The colors denote whether the correlation is positive (closer to 1; purple) or negative (closer to −1; orange) between the bacteria and the efficiency parameters. Color intensity is proportional to Spearman’s rank correlation values. Spearman’s test, *; *p* < 0.05; **; *p* < 0.01.

Spearman’s correlation analysis indicated correlations among several characteristics using different levels of DGP. Twenty-four indexes related to GP showed high positive or negative correlations, indicating that major bacteria were closely related to the apparent digestibility of nutrients, nitrogen metabolism, blood antioxidant indexes, and rumen fermentation parameters (Figure 4). In the rumen (Figure 4A), *Succiniclasticum* (class Negativicutes) was positively associated with ether extract digestibility (EED) (*p* < 0.01), dry matter digestibility (DMD) (*p* < 0.05), urinary nitrogen (*p* < 0.05), CAT (*p* < 0.01), and T-AOC (*p* < 0.05) and negatively correlated with acetate (*p* < 0.05), isobutyrate (*p* < 0.05), butyrate (*p* < 0.05), the acetate-to-propionate ratio (*p* < 0.01), and total VFA (*p* < 0.05). *Fibrobacter* (phylum Fibrobacterota) was negatively correlated with EED (*p* < 0.05).

## 4. Discussion

The condensed tannins and lignins in GP emerge as pivotal factors influencing digestive processes [27,28]. However, recent studies have demonstrated that CT from various sources and with distinct chemical structures exhibit clear dose effects but above a certain threshold can cause negative effects [29]. Total tannins can be beneficial bioactive compounds in ruminant diets when included at levels below 3% of dietary dry matter [30]. In this study, the total tannin content within the 20% dried grape pomace diet did not surpass 2 g/kg. Despite this, the observed ADG and apparent digestibility of the beef cattle were lower compared to those in the control group. These findings suggest that the limitations observed in the diet were attributed to more than just the tannin content in the GP. Lignin acts as a physical barrier against rumen microorganisms, inhibiting the degradation of cell walls. This hindrance in the digestive processes results in a decrease in the apparent digestibility of nutrients [31,32,33]. Abarghuei et al. [32] observed that even after eliminating the influence of CT, the apparent digestibility of nutrients remained significantly reduced. This finding underscores that lignin independently exerts a detrimental impact on the growth performance of beef cattle. In this study, the lignin content (ADL = 7.88% DM) observed in the G20 group may have negatively influenced the growth performance of the beef cattle. Consequently, it is imperative to consider both the tannin and lignin content when incorporating GP into the diet.

Upon entering the rumen, dietary lipids transform processes such as lipolysis and fatty acid biohydrogenation (BH). A portion is hydrolyzed by microbial lipases, yielding nutrients such as glycerol for bacterial utilization [34]. The remaining lipids proceed through the rumen and are digested and absorbed in the small intestine. The microbial results of this experiment showed that *Butyrivibrio* (Figure 3) was the dominant genus in group G0, which was shown to be involved in further steps of BH after lipolysis [10]. After the addition of GP, the dominant genera changed to *unclassified-Christensenellaceae* and *Pantoea* in the rumen. Additionally, research indicates that tannins can diminish the release of free fatty acids by inhibiting lipase activity, and they can impede lipid digestion by binding to bile salts [35]. In this experiment, the EED of the 20% group exhibited a decrease, aligning with the findings observed by Molosse [36]. This decline may be attributed to the inhibitory effects of the tannins in GP on lipid absorption.

Our observations revealed a linear decrease in urinary nitrogen with an increasing addition of GP, a phenomenon likely attributed to the concurrent rise in tannin content. This correlation suggests the direct impact of the tannin content in GP on ruminal protein metabolism. Relevant studies indicate that supplementing feed with the appropriate tannins can alter the nitrogen pathway, facilitating nitrogen transfer from the urine to the feces [37,38]. This, in turn, contributes to a reduction in the loss of highly volatile forms of nitrogen. On the one hand, tannins bind with a portion of the protein in the rumen, leading to decreased utilization of dietary protein within the rumen. On the other hand, tannins interact with proteins, causing a shift in the location of nitrogen metabolism and digestion. Additionally, this diminishes microbial enzyme activity, thereby slowing down the growth rate of hydrolytic bacteria and reducing the production of ruminal NH_3_-N. Rumen microorganisms absorb excess nitrogen into NH_3_-N and convert it into urea and then excrete it through the urine or circulate it in the intestine. Consequently, the interaction between tannins and proteins in the rumen can mitigate the conversion of nitrogen in the diet, reducing the urinary nitrogen content [39]. Therefore, the utilization of GP holds significant potential for promoting the green and low-carbon development of beef cattle. Previous studies have established a positive correlation between fecal nitrogen excretion and urinary nitrogen and nitrogen intake [40]. The nitrogen in feces predominantly exists as organic nitrogen, which exhibits lower volatility. Conversely, the nitrogen in urine primarily exists in the form of urea, which undergoes rapid oxidation into NO_3_^−^ and nitrite (N_2_O^−^) through soil nitrification [41]. This process generates N_2_O, an intermediate product recognized as a potent greenhouse gas. In contrast, the addition of GP allows for a reduction in the degradation and deamination rate of dietary protein in the rumen [37,42,43]. This alteration in the nitrogen pathways helps mitigate N_2_O emissions, thereby contributing to the goal of reducing greenhouse gas emissions during both production and manure treatment.

During times of oxidative stress, cells generate a significant amount of reactive oxygen species (ROS). This increase in ROS levels can lead to inflammation, prompting the body to rely on antioxidant enzymes and antioxidants for the purpose of counteracting the sudden rise in ROS. Examples include SOD and CAT, which act to safeguard cells against the damage caused by ROS [44,45]. High-concentrate diets promote oxidative stress in beef cattle, so we can promote increases in endogenous antioxidants (SOD and CAT) by supplementing exogenous antioxidant molecules to offset the ROS of beef cattle. SOD is the first line of defense, catalyzing superoxides to produce oxygen and hydrogen peroxide, while CAT neutralizes the decomposition of hydrogen peroxide into oxygen and water [46]. Veras [47] showed that tannic acid in feed promoted an increased CAT concentration in mice. Feed including 10% GP was found to increase the concentration of CAT, similar to the results of previous studies. However, a high proportion of GP in the diet did not improve the antioxidant levels in beef cattle. This could be due to the pro-oxidation effect of a high proportion of polyphenol compounds in beef cattle. Studies have shown that the polyphenols in bilberry extract lead to apoptosis in cancer cells by increasing intracellular ROS levels [48]. Combined with heatmap analysis, although the SOD concentration in the serum did not significantly change in the 20% group in this experiment, the abundance of *Bifidobacterium* decreased after they were fed 20% GP, and *Bifidobacterium* was negatively correlated with T-AOC. Therefore, we speculated that 20% added GP may be the counterbalance of antioxidant and pro-oxidation effects in the body, but further studies are needed to confirm this.

The digestion and utilization of feed in ruminants are primarily linked to the capacity of the rumen microbial community to convert potentially digestible feed into metabolizable nutrients [49]. Rumen microorganisms play a dual role, satisfying the majority of the nutritional requirements for ruminants and accounting for up to 90% of their metabolic needs [50,51]. The results of this experiment indicate that the diversity of rumen microorganisms in beef cattle remains unaltered. This suggests that including GP in the diet does not induce microbial disorders in the rumen of beef cattle during the fattening period. Generally, the rumen exhibits the highest proportion of Bacteroidetes, followed by Firmicutes [52,53,54]. It is worth noting that the body weight of beef cattle in the 20% group decreased in this trial, suggesting a potential association with alterations in rumen species distribution (Figure 1) and the characteristics of diet (Table 2). Bacteroidetes have exhibited precise mechanisms of orchestrating the expression of polysaccharide-degrading enzymes, thereby regulating carbohydrate digestion and absorption at the genetic level [55,56]. Firmicutes play a role in promoting the digestion and absorption of proteins within animal organisms. Additionally, they can produce free enzymes, cellulosomes, or multi-enzyme complexes during the process of fiber digestion [56]. This collaborative action facilitates the degradation of polysaccharides and the production of lactic acid and butyric acid, which can be utilized by the liver and muscle tissues [57]. Furthermore, an investigation into the gastrointestinal tract of humans and mice indicated a positive association between the Firmicutes/Bacteroidetes ratios and obesity. The study suggested that Firmicutes/Bacteroidetes influence fat storage by regulating the energy absorption efficiency [58,59]. This aligns with our observation that the relative abundance of Firmicutes in the G20 group was diminished, consequently impacting the energy absorption from the diet in the beef cattle and reducing the FCR.

Additionally, the proportion of added GP will impact the composition of the rumen microbiota. Interestingly, including 20% GP in the diet increased the abundance of Fibrobacterium in the rumen. This observation may signify a metabolic strategy beef cattle employ to adapt to higher-lignocellulose diets. *F. succinogenes* belongs to Fibrobacterota. The protein in the outer membrane that *Succinogenes* possesses has a distinctive ability to bind cellulose, which plays a crucial role in fiber degradation [56,60,61]. An elevated lignocellulose content promotes the growth of Fibrobacteria, facilitating their increased production of VFA, essential to the host animal’s maintenance, reproduction, and growth [62]. However, the proliferation of fibrolytic bacteria may increase the acetate/propionate ratio, potentially negatively affecting the efficiency of dietary energy utilization. In this study, adding 10% GP promoted the enrichment of *Succiniclasticum*. *Succiniclasticum*, classified within the Negativicutes class, is the primary bacterium engaged in the succinic acid pathway, producing propionic acid. The relative abundance of *Succiniclasticum* showed a positive correlation with propionic acid production [63]. Hence, the addition of 10% GP can partially facilitate an increase in the acetate-to-propionate ratio in beef cattle, thereby contributing to a balance in the impact of *Succinogenes*. The heatmap showed that the antioxidant index (CAT and T-AOC) had a significant positive correlation with *Succiniclasticum*, indicating that the addition of 10% GP is conducive to improving the genes of *Succiniclasticum*, thus improving the health of beef cattle.

Nevertheless, when the GP is increased to 20%, the stability of the microbial structure in the rumen diminishes. This could be attributed to the excessive addition of GP, disrupting the composition of the rumen microbial community and causing alterations in the keystone taxa of the rumen microorganisms. Keystone taxa significantly influence microbial communities and functions, irrespective of the abundance of microbiota across spatial and temporal dimensions [64]. Katharine et al. [65] argue that although positive interactions improve the overall metabolic efficiency, they reduce ecological stability. Thus, a higher neg/pos ratio indicates weaker microbial competition and a more stable network structure. In combination with other indicators, the network diagram became more complex with an increase in GP, but the microbial network diagram did not become more stable with an increase in GP. The stability of the microbial network and the antioxidant indexes both increased first and then decreased. These results suggested that the stability of the rumen microbiota may be affected by the antioxidant properties of GP after feeding, thus affecting the overall metabolic efficiency, which may also explain the decrease in body weight and the apparent digestibility of nutrients observed in G20.

In this study, rectum Firmicutes showed a relative abundance increase with an elevated GP intake, while Bacteroidetes linearly decreased. It is a pity that there is a paucity of studies on the rectal microbiota of ruminants. A study of human feces revealed a correlation between this trend and bacteria aiding the further breakdown of tannins into phenolic acids [66]. However, whether the breakdown products in beef cattle align with those in the human gastrointestinal tract remains to be explored.

We observed an increase in the relative abundance of *Lachnospiraceae* after feeding with 20% GP, potentially attributable to an elevation in polyphenols in the diet. Studies have demonstrated that polyphenols can modulate the relative abundance of *Lachnospiraceae* in the rectum, aligning with the findings of this study [67]. Moreover, *Lachnospiraceae* have been associated with maintaining healthy immune homeostasis and preventing human inflammatory diseases [68]. Including GP may play a role in modulating hindgut health in beef cattle. In this study, the relative abundance of UCG_004 decreased after adding 20% GP. UCG_004, a member of *Erysipelatoclostridiaceae*, is a potential pathogen associated with colon diseases [69]. Previous reports have indicated that polyphenols can enhance ruminants’ antioxidant activity by constraining pathogenic bacteria’s growth [15,70], and the results of this study suggest the potential that adding a certain proportion of GP may contribute to reducing inflammation in diseases.

## 5. Conclusions

In the current study, the optimal proportion of GP added to the feed of Angus bulls during the fattening period appeared to be 10%. At this ratio, there was no observed negative impact on the growth performance and apparent digestibility of the beef cattle, while the urinary nitrogen emissions showed a reduction, and the antioxidant activity was increased. The predominant rumen bacteria in G10 was *Succiniclasticum*, which was positively correlated with the antioxidant indexes of the beef cattle. Adding 10% GP resulted in more favorable microbial community complexity and stability. These findings offer valuable insights into the potential benefits of incorporating GP into the diet of ruminants.

## Figures and Tables

**Table 1:** Feed ingredient proportions of experimental diets. (DM basis, %).

Items	G0	G10	G20
Ingredient composition			
Corn silage	26.08	13.05	0.00
Rice straw	11.61	11.61	11.60
DGP	0.00	10.44	20.88
Ground corn	39.91	46.93	53.69
Rapeseed meal	7.22	6.69	6.49
Soybean meal	2.16	2.17	2.14
Wheat bran	7.82	3.91	0.00
NaHCO_3_	1.11	1.11	1.11
NaCl	0.37	0.37	0.37
MgO	0.44	0.44	0.44
Premix ^1^	3.28	3.28	3.28
Total	100	100	100

^1^ One kilogram of premix contained the following: VA 120,000–200,000 IU; VE ≥ 550 IU; D-Biotin ≥ 0.3 mg; VD3 15,000–60,000 IU; VB3 ≥ 350 mg; Cu 0.16–0.5 g; Mn 0.6–2.4 g; Se 1.6–10 mg; Ca 100–200 g; Fe 0.8–8.4 g; Zn 1.5–3.0 g; I 4–20 mg; NaCl 100–200 g; TP ≥ 20 g. DGP, dried grape pomace.

**Table 2:** Chemical composition and fatty acid levels of basal diets (DM basis).

Items	DCP ^1^	G0	G10	G20
Chemical composition				
CP; %	11.69	11.89	11.44	12.02
EE; %	9.80	2.13	2.28	2.39
NDF; %	42.72	26.69	27.83	29.58
ADF; %	40.65	14.38	16.49	20.06
ADL; %	21.24	2.43	3.88	7.88
Ash; %	11.11	8.98	9.79	10.92
TT; g/kg	7.98	1.46	1.71	1.86
CT; g/kg	2.49	1.55	1.74	1.84
TDN ^2^; %DM	65.08	74.89	74.20	73.12
DE ^2^; MJ/kg DM	9.55	12.51	12.30	11.98
Fatty acids (g/100 g of total fatty acid)				
14:0	0.06	0.08	0.07	0.07
16:0	7.42	9.11	8.95	8.62
18:0	3.47	0.93	1.75	1.82
18:1n-9	10.99	12.89	12.93	12.97
18:2n-6	55.93	28.16	34.31	37.05

^1^ was adapted from Li et al. [20]. ^2^ TDN (total digestible nutrients) and DE (digestible energy) were calculated according to Wang (2016)’s [21] model. Note. G0, the control group; G10, the 10% dried grape pomace treatment group; G20, the 20% dried grape pomace treatment group; DGP, dried grape pomace; DM, dry matter; CP, crude protein; EE, ether extract; NDF, neutral detergent fiber; ADF, acid detergent fiber; ADL, acid detergent lignin; TT, total tannins; CT, condensed tannins.

**Table 3:** Effects of dried grape pomace at different levels on growth performance in Angus bulls.

Items	G0	G10	G20	SEM	*p*-Value	Linear	Quadratic
Initial live weights; kg	580.40	580.40	580.90	7.800	1.000	0.980	0.988
Final live weights; kg	749.85	746.75	712.80	8.141	0.106	0.054	0.376
ADG; kg	1.47 ^a^	1.40 ^a^	1.14 ^b^	0.052	0.014	0.006	0.354
FCR	9.01 ^b^	9.37 ^b^	11.62 ^a^	0.427	0.015	0.007	0.267

^a,b^ Means within a row with different superscripts differ (*p* < 0.05). G0, the control group; G10, the 10% dried grape pomace treatment group; G20, the 20% dried grape pomace treatment group; SEM, standard error of the mean; ADG, average daily gain; FCR, feed conversion rate—the DMI/ADG ratio.

**Table 4:** Effects of dried grape pomace at different levels on nutrients’ apparent digestibility in Angus bulls.

Items	G0	G10	G20	SEM	*p*-Value	Linear	Quadratic
Apparent digestibility (%)							
DM	67.48	65.89	62.73	1.526	0.449	0.218	0.810
CP	67.01	60.80	59.93	1.760	0.206	0.105	0.470
EE	74.98 ^a^	73.81 ^a^	57.60 ^b^	2.658	0.007	0.005	0.134
NDF	50.43	48.23	44.71	2.073	0.541	0.276	0.883
ADF	43.64	43.04	39.01	2.189	0.659	0.405	0.721
Nitrogen metabolism (g/d)							
Nitrogen intake	228.85	233.85	239.69	-	-	-	-
Fecal nitrogen	81.94	89.40	96.64	4.434	0.432	0.200	0.991
Urinary nitrogen	88.15	74.76	66.64	4.108	0.100	0.034	0.751
Nitrogen retention	66.18	72.10	83.36	6.160	0.540	0.288	0.840
Nitrogen utilization (%)	28.68	31.99	29.51	2.079	0.805	0.880	0.523

^a,b^ Means within a row with different superscripts differ (*p* < 0.05). G0, the control group; G10, the 10% dried grape pomace treatment group; G20, the 20% dried grape pomace treatment group; SEM, standard error of the mean; DM, dry matter; CP, crude protein; EE, ether extract; NDF, neutral detergent fiber; ADF, acid detergent fiber.

**Table 5:** Effects of dried grape pomace at different levels on blood antioxidant indexes of Angus bulls.

Items	G0	G10	G20	SEM	*p*-Value	Linear	Quadratic
CAT, U/mL	13.22 ^b^	29.39 ^a^	7.25 ^b^	3.019	0.004	0.350	0.002
T-AOC, Trolox Mm	1.00 ^ab^	1.01 ^a^	0.96 ^b^	0.008	0.041	0.057	0.082
SOD, U/mL	13.77	14.34	14.06	0.260	0.681	0.654	0.456
MDA, nmol/mL	3.55	2.15	2.90	0.287	0.133	0.343	0.080
ROS, IU/mL	673.21	869.22	778.75	37.751	0.579	0.501	0.407

^a,b^ Means within a row with different superscripts differ (*p* < 0.05). G0, the control group; G10, the 10% dried grape pomace treatment group; G20, the 20% dried grape pomace treatment group; SEM, standard error of the mean; CAT, catalase; T-AOC, total antioxidant capacity; SOD, superoxide dismutase; MDA, malondialdehyde; ROS, reactive oxygen species.

**Table 6:** Effects of dried grape pomace at different levels on rumen fermentation parameters in Angus bulls.

Items	G0	G10	G20	SEM	*p*-Value	Linear	Quadratic
pH	6.36	6.57	6.42	0.048	0.181	0.573	0.080
Ammonia-N; mg/dL	5.09	4.63	4.04	0.216	0.177	0.087	0.872
VFA; mmol/L							
Acetate; mmol/L	85.78	82.41	88.60	3.921	0.823	0.777	0.581
Propionate; mmol/L	26.69	26.52	21.55	1.225	0.151	0.087	0.348
Isobutyrate; mmol/L	0.94	0.96	1.11	0.051	0.332	0.179	0.537
Butyrate; mmol/L	16.67	14.73	15.69	0.904	0.698	0.670	0.468
Isovalerate; mmol/L	3.08	2.45	2.75	0.158	0.283	0.401	0.178
Valerate; mmol/L	1.58	1.36	1.26	0.064	0.121	0.046	0.650
Acetate-to-propionate ratio	3.27 ^b^	3.22 ^b^	4.27 ^a^	0.175	0.015	0.014	0.102
Total VFA; mmol/L	134.73	128.43	130.96	5.720	0.909	0.796	0.728

^a,b^ Means within a row with different superscripts differ (*p* < 0.05). G0, the control group; G10, the 10% dried grape pomace treatment group; G20, the 20% dried grape pomace treatment group. SEM, standard error of the mean.

## Data Availability

The original manuscript of this study is included in the article, and further information is available upon reasonable request to the corresponding author.

## Supplementary Materials

The following supporting information can be downloaded at https://www.mdpi.com/article/10.3390/antiox13040412/s1. Table S1. Effects of dried grape pomace at different levels on bacterial composition in rumen and rectum at the phylum level (relative abundance > 0.01%) of beef cattle. Table S2. Effects of dried grape pomace at different levels on bacterial composition in rumen and rectum at the family level (relative abundance > 0.1%) of beef cattle. Table S3. Effects of dried grape pomace at different levels on bacterial composition in rumen and rectum at the genus level (relative abundance > 0.5%) of beef cattle.
