# Supplementary material for: Effects of Grape Pomace on Growth Performance, Nitrogen Metabolism, Antioxidants, and Microbial Diversity in Angus Bulls
Source: Antioxidants (Basel). 2024 Mar 28;13(4):412. doi: 10.3390/antiox13040412 (PMC11047470; doi:10.3390/antiox13040412)
Supplement: Supplementary file 1 [file antioxidants-13-00412-s001.zip › antioxidants-2919397-supplementary.pdf]

Supplementary Materials:

Supplementary Table S1. Effects of dried grape pomace at different levels on bacterial composition in rumen and rectum at the phylum level (relative abundance > 0.01%) of beef cattle.

Items	G0	G10	G20	SEM	*P*-value	Linear	Quadratic
Rumen							
Actinobacteriota	73.4	50.0	21.0	12.101	0.213	0.082	0.912
Bacteroidota	18340.8	18384.9	18674.4	740.161	0.982	0.860	0.940
Cyanobacteria	20.0	19.5	11.7	6.430	0.849	0.613	0.797
Desulfobacterota	171.2	171.1	128.5	16.925	0.510	0.317	0.563
Fibrobacterota	191.4	401.6	453.8	63.113	0.203	0.094	0.551
Firmicutes	15256.7	15993.1	12696.4	847.579	0.257	0.222	0.265
Patescibacteria	666.1	693	631.3	46.206	0.870	0.768	0.665
Proteobacteria	3489.4	2542.9	5202.5	612.207	0.203	0.252	0.167
Spirochaetota	118.7	64.2	419.5	84.788	0.186	0.149	0.252
Verrucomicrobiota	202	217.2	240.5	29.809	0.876	0.613	0.951
Rectum							
Actinobacteriota	312.3[a]	277.6[a]	38.4[b]	44.512	0.018	0.009	0.236
Bacteroidota	12084.9	10744.1	9158.3	545.470	0.086	0.029	0.912
Cyanobacteria	167.4	54.1	52.0	48.372	0.554	0.345	0.597
Desulfobacterota	16.1[a]	20.6[a]	8.5[b]	1.897	0.025	0.082	0.031
Firmicutes	22211.5[b]	23137.9[b]	26445.4[a]	573.259	0.003	0.001	0.253
Patescibacteria	246.3	186.0	245.7	40.687	0.797	0.995	0.505
Proteobacteria	3322.3	4147.7	2628.2	394.136	0.299	0.474	0.168
Spirochaetota	218.5	0.3	0.0	49.935	0.118	0.073	0.292

[a; b] Means within a row with different superscripts differ (*P*<0.05).
G0: the control group, G10: the 10% dried grape pomace treatment group, G20: the 20% dried grape pomace treatment group, SEM = standard error of the mean.

Supplementary Table S2. Effects of dried grape pomace at different levels on bacterial composition in rumen and rectum at the family level (relative abundance > 0.1%) of beef cattle.

Family level	G0	G10	G20	SEM	*P*-value	Linear	Quadratic
Rumen							
Acidaminococcaceae	2884.2[a]	3360.7[a]	1734.6[b]	222.034	0.005	0.019	0.014
Bacteroidales_RF16_group	2105.5	1701.6	1458.4	238.988	0.552	0.285	0.877
Christensenellaceae	619.1	749.4	598.2	100.611	0.813	0.935	0.527
Clostridiaceae	28.4	26.7	11.5	5.860	0.447	0.252	0.594
F082	1148.4	1547.5	2829.1	338.247	0.103	0.043	0.524
Lachnospiraceae	4100	3822.2	3488.4	363.154	0.801	0.509	0.972
Muribaculaceae	833.5	744.7	506.5	77.146	0.206	0.088	0.644
Oscillospiraceae	2717.8	3038.3	2579.2	166.044	0.528	0.739	0.284
Prevotellaceae	11613.1	11593.9	9861.9	625.946	0.438	0.266	0.527
Rikenellaceae	1888.4	2087.9	3182.3	294.020	0.155	0.074	0.465
Ruminococcaceae	1589.3	1328.1	1202.6	140.078	0.533	0.275	0.823
Selenomonadaceae	497.1	728.0	548.1	60.009	0.264	0.728	0.114
Succinivibrionaceae	3419.3	2418.5	5023.9	615.267	0.224	0.287	0.170
Rectum							
Acidaminococcaceae	813.7	817.5	566.5	51.523	0.070	0.047	0.225
Bacteroidaceae	1170.7	1230.2	1199.6	129.342	0.984	0.930	0.875

Bacteroidales_RF16_group	48.2	24.3	76.2	21.204	0.623	0.601	0.415
Christensenellaceae	270.1	345.0	513.3	49.827	0.123	0.047	0.649
Clostridiaceae	736.3	953.8	1063.7	107.020	0.461	0.226	0.816
Lachnospiraceae	8093.7[b]	8774.8[b]	10957.6[a]	474.557	0.030	0.012	0.420
Muribaculaceae	1904.5	1873.0	1469.4	132.180	0.337	0.188	0.511
Oscillospiraceae	5924.7	6851.2	7320.4	281.968	0.119	0.044	0.693
Prevotellaceae	5369.1	4364.2	3127.8	427.956	0.098	0.033	0.894
Rikenellaceae	2441.0	2463.9	2169.1	312.516	0.918	0.734	0.818
Ruminococcaceae	1446.6	1046.7	916.8	134.068	0.250	0.113	0.634
Selenomonadaceae	172.8	237.1	136.1	32.391	0.451	0.649	0.242
Succinivibrionaceae	2769.1	3720.8	2152.7	403.570	0.288	0.534	0.149

[a; b] Means within a row with different superscripts differ (*P*<0.05).
G0: the control group, G10: the 10% dried grape pomace treatment group, G20: the 20% dried grape pomace treatment group, SEM = standard error of the mean.

Supplementary Table S3. Effects of dried grape pomace at different levels on bacterial composition in rumen and rectum at the genus level (relative abundance > 0.5%) of beef cattle.

Family level	G0	G10	G20	SEM	*P*-value	Linear	Quadratic
Rumen							
Alloprevotella	18.4	16.5	55.2	16.211	0.563	0.369	0.566
Clostridium_sensu_stricto_1	28.4	26.7	11.5	5.860	0.447	0.252	0.594
NK4A214_group	1768.7	1855.3	1252.5	153.806	0.230	0.174	0.291
Prevotella	8932.3	9441.9	7879.1	626.663	0.600	0.506	0.450
Rikenellaceae_RC9_gut_group	1881.8	2069.9	3164.7	294.459	0.159	0.077	0.460
Ruminococcus	1126.9	845.9	726.0	97.564	0.233	0.099	0.695
Succinivibrio	85.1	90.8	129.4	27.174	0.782	0.522	0.783
Succinivibrionaceae_UCG_002	1318.2	1052.0	2587.4	376.934	0.211	0.171	0.260
UCG_005	225.0	306.9	297.0	31.860	0.535	0.370	0.508
unclassified_Bacteroidales_RF16_group	1915.8	1598.7	1353.6	223.971	0.606	0.323	0.941
unclassified_F082	833.6	1061.8	995.8	123.122	0.752	0.604	0.587
unclassified_Lachnospiraceae	1139.3	758.0	784.4	167.555	0.600	0.403	0.577
unclassified_Muribaculaceae	38.3	28.9	47.2	7.645	0.636	0.645	0.409
unclassified_Prevotellaceae	933.7[a]	795.4[ab]	512.7[b]	70.425	0.039	0.013	0.603
unclassified_Succinivibrionaceae	627.3[b]	474.2[b]	1758.9[a]	220.851	0.028	0.029	0.103
uncultured_rumen_bacterium	2622.2[ab]	2960.0[a]	2164.8[b]	130.996	0.039	0.133	0.035
Succiniclasticum	2884.2[a]	3360.3[a]	1734.4[b]	222.041	0.005	0.019	0.014
Rectum							
Agathobacter	980.7	895.2	868.6	92.192	0.882	0.634	0.885
Alloprevotella	1389.5	1016.7	719.8	150.924	0.196	0.074	0.904
Bacteroides	1170.7	1230.2	1199.6	129.342	0.984	0.930	0.875
Clostridium_sensu_stricto_1	700.3	934.4	963.2	100.888	0.522	0.302	0.639
NK4A214_group	144.5	173.6	354.3	39.320	0.056	0.027	0.338
Prevotella	408.1	231.2	173.8	47.492	0.108	0.044	0.540
Prevotella_9	2413.6	1838.1	1115.8	268.460	0.141	0.050	0.895
Rikenellaceae_RC9_gut_group	1577.3	2009.0	1616.4	221.882	0.695	0.944	0.399
Ruminococcus	136.1	134.1	184.1	30.114	0.758	0.531	0.695
Succinivibrio	2191.5	3615.6	1999.5	373.850	0.157	0.830	0.058
UCG_005	5139.9	6127.0	6279.7	272.953	0.183	0.091	0.465
unclassified_Lachnospiraceae	5018.6	4896.0	6349.9	286.107	0.066	0.053	0.177
unclassified_Muribaculaceae	1562.0	1640.6	1217.4	118.560	0.311	0.242	0.324
unclassified_Prevotellaceae	939.1	830.1	466.7	109.116	0.183	0.080	0.577

uncultured_rumen_bacterium	194.0	60.4	116.3	38.135	0.369	0.412	0.251
Succiniclasticum	3.0	0.7	5.3	0.946	0.139	0.312	0.085

[a; b] Means within a row with different superscripts differ (P<0.05).

G0: the control group, G10: the 10% dried grape pomace treatment group, G20: the 20% dried grape pomace treatment group, SEM = standard error of the mean.
